# Nasal Dilators (Breathe Right Strips and NoZovent) for Snoring and OSA: A Systematic Review and Meta-Analysis

**DOI:** 10.1155/2016/4841310

**Published:** 2016-12-13

**Authors:** Macario Camacho, Omojo O. Malu, Yoseph A. Kram, Gaurav Nigam, Muhammad Riaz, Sungjin A. Song, Anthony M. Tolisano, Clete A. Kushida

**Affiliations:** ^1^Tripler Army Medical Center, Division of Otolaryngology, Sleep Surgery and Sleep Medicine, 1 Jarrett White Rd, Tripler AMC, Honolulu, HI 96859, USA; ^2^Uniformed Services University of the Health Sciences, 4301 Jones Bridge Road, Bethesda, MD 20814, USA; ^3^Tripler Army Medical Center, Division of Otolaryngology-Head & Neck Surgery, Tripler AMC, Honolulu, HI 96859, USA; ^4^Clay County Hospital, 911 Stacy Burk Drive, Flora, IL 62839, USA; ^5^Sleep Disorders Center, Sunnyside Community Hospital, 1016 Tacoma Avenue, Sunnyside, WA 98944, USA; ^6^Department of Psychiatry and Behavioral Sciences, Sleep Medicine Division, Stanford Hospital and Clinics, Redwood City, CA 94063, USA

## Abstract

*Objective*. To systematically review the international literature for studies evaluating internal (NoZovent) and external (Breathe Right Strips) nasal dilators as treatment for obstructive sleep apnea (OSA).* Study Design*. Systematic review with meta-analysis.* Methods*. Four databases, including PubMed/MEDLINE, were searched through September 29, 2016.* Results*. One-hundred twelve studies were screened, fifty-eight studies were reviewed, and fourteen studies met criteria. In 147 patients, the apnea-hypopnea index (AHI) was reported, and there was an improvement from a mean ± standard deviation (M ± SD) of 28.7 ± 24.0 to 27.4 ± 23.3 events/hr, *p* value 0.64. There was no significant change in AHI, lowest oxygen saturation, or snoring index in OSA patients when using nasal dilators. However, a subanalysis demonstrated a slight reduction in apnea index (AI) with internal nasal dilators (decrease by 4.87 events/hr) versus minimal change for external nasal dilators (increase by 0.64 events/hr).* Conclusion*. Although nasal dilators have demonstrated improved nasal breathing, they have not shown improvement in obstructive sleep apnea outcomes, with the exception of mild improvement in apnea index when internal nasal dilators were used.

## 1. Introduction

The nose is composed of both internal and external structures. Although the internal structures of the nose (i.e., turbinates [1], septum [2]) do not generally move in a dynamic fashion, they can become edematous with associated symptoms of congestion and obstruction. It is known that the nose may contribute to snoring [3] and obstructive sleep apnea (OSA) when congested or obstructed. There are many treatments for OSA, to include medical management with positive airway pressure devices [4], oral appliances, and myofunctional therapy [5]. Nasal therapies to help treat OSA include nasopharyngeal airway stenting devices [6], nasal expiratory positive airway pressure devices (Provent) [7], and nasal surgery [8, 9].

The simple act of changing from the upright to the supine position has been shown to reduce upper airway volume by approximately 33% in OSA patients [10]. Given that the nasal cavity is upstream from the collapsible soft tissues of the upper airway, the nasal cavity directly influences the downstream airflow. Moreover, when the nasal cavity's cross-sectional area increases by 10%, there is a corresponding 21% increase in nasal airflow [11].

Internal (NoZovent) and external (Breathe Right Strips) nasal dilators have been studied in the treatment of OSA for over twenty years, but to date there are no published meta-analyses of these interventions. Therefore, the objective of this study was to systematically review the international literature, without regard to language, for studies evaluating the pretreatment and treatment data of both internal and external nasal dilators as treatment for OSA and to then use the data to perform a meta-analysis.

## 2. Methods

Three authors (M. C., O. O., and M. R.) independently searched PubMed/MEDLINE, Scopus, Embase, Google Scholar, and The Cochrane Library for studies, through September 29, 2016.

### 2.1. Search Strategy

Specific phrases, keywords, and MeSH terms were tailored to each database as appropriate. An example of a search strategy used in PubMed/MEDLINE includes (((“instrumentation” [Subheading]) AND (“Nasal Obstruction” [Mesh])) AND “Sleep Apnea Syndromes”) OR ((“Dilatation” [Mesh] AND (“instrumentation” [Subheading])) AND (((“Sleep Apnea, Obstructive” [Mesh]) OR (“Snoring” [Mesh]) AND “Dilatation” [Mesh])) AND (“Nasal Obstruction” [Mesh])) OR ((nasal dilator [tiab]) AND ((sleep apnea [All Fields] OR (sleep apnoea [All Fields]))) OR (nasal dilatation^*∗*^ AND sleep apnea^*∗*^) OR (nasal dilatation^*∗*^ AND snoring^*∗*^)).

### 2.2. Study Selection

The inclusion criteria were as follows: (1)* patients*: adults ≥18 years old who have OSA, (2)* intervention*: nasal dilators, (3)* comparison*: sleep study data pretreatment and treatment, (4)* outcome*: sleep study parameters including apnea-hypopnea index (AHI), apnea index (AI), oxygen saturations, and sleepiness, and (5)* study designs*: all designs and all languages.

Studies reporting only qualitative outcomes and those containing a diagnosis of central sleep apnea were excluded.

### 2.3. Data Abstraction

Articles were independently reviewed and logged by two authors (M. C. and Y. A. K.). Patient sleep study data (i.e., lowest oxygen saturation (LSAT), mean oxygen saturation (MSAT), AHI, AI, respiratory disturbance index (RDI)), ages, body mass index (BMI), and quantitative sleepiness data (e.g., Epworth sleepiness scale (ESS) [12]) were collected. The National Institute for Health and Clinical Excellence (NICE) quality assessment tool [13] was utilized to evaluate each study; see Table 1.

### 2.4. Statistical Analysis

Our null hypothesis is that no difference exists in the outcomes when comparing pretreatment and treatment data. Statistical calculations were performed with STATA 14.1 (StataCorp, College Station, Texas, USA) and Review Manager Software (REVMAN) version 5.3 (Copenhagen: The Nordic Cochrane Centre: The Cochrane Collaboration, 2014). Means (M) and standard deviations (SD) were calculated with STATA 14.1. Mean differences (MD), standardized mean differences (SMD), SD, and 95% confidence intervals [95% CI] were calculated with the REVMAN program. Statistical significance was defined as a *p* value < 0.05. Random effects modeling was calculated. Cohen's guidelines [14] were followed when assigning either a small, medium, or large magnitude of effect. If a study reported the means but did not report the standard deviation, then the weighted average from the other studies in the meta-analysis was utilized (consistent with a technique from a previously published meta-analysis) [15].

Heterogeneity was evaluated using the Cochrane *Q*-statistic (*Q*-statistic), with significance defined as a *p* value ≤ 0.10 [16]. The *I* ^2^ was used to evaluate for inconsistency [17].

## 3. Results

One-hundred twelve studies were potentially relevant, fifty-eight studies were downloaded in full text form, twenty-four studies had data, and fourteen studies [18–31] had quantitative polysomnography data; see Table 2. Internal nasal dilators were used in five studies and external nasal dilator strips were used in nine studies.

### 3.1. Polysomnography Scoring Criteria

There was significant heterogeneity in polysomnographic scoring criteria among studies. Specifically, Amaro et al. defined a hypopnea as a drop of >50% in the scoring signals with ≥3% oxygen desaturation. Bahammam et al. defined a hypopnea as ≥3% oxygen desaturation for 6 seconds or more, whereas Hoijer et al. used a ≥4% oxygen desaturation. Pevernagie et al. scored hypopneas when there was a ≥2% oxygen desaturation for at least 10 seconds. Finally, Redline et al. defined hypopneas as a discernable change in airflow lasting at least 10 seconds corresponding to a 2.5% decrease in O_2_ saturation or resulting in arousal. The remaining studies either stated that they followed standard polysomnography scoring criteria or did not mention the scoring criteria used.

### 3.2. The Effect of Nasal Dilators on Sleep Stages

The total percentage of total sleep time (TST) spent in sleep stages N1, N2, and N3 and rapid eye movement (REM) were evaluated. The respective pretreatment and treatment values for percent TST spent in each stage were N1/N2 = 62.8% and 63.8%; N3 = 20.2 ± 14.6% and 17.6 ± 15.6% (two-tailed *p* value = 0.2183); REM = 17.0 ± 7.3% and 18.6 ± 6.1% (two-tailed *p* value = 0.0894). Overall, there was no statistically significant or clinically significant difference in the sleep architecture.

### 3.3. Apnea-Hypopnea Index and Respiratory Disturbance Index

**Figure 1:**
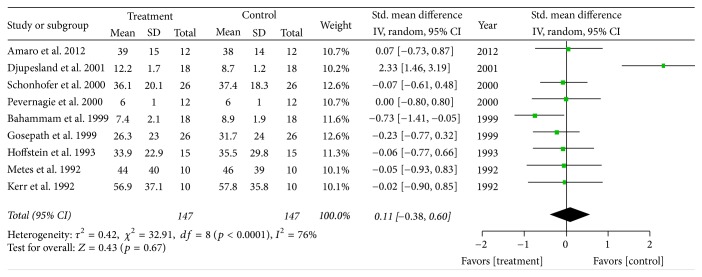
Standardized mean difference for apnea-hypopnea index. The overall standardized mean difference is 0.11 [−0.38,0.60] corresponding to a small effect.

Ten studies reported AHI outcomes and nine provided M ± SD. There were a total of 147 patients who had a combined pretreatment and treatment M ± SD of 28.7 ± 24.0 and 27.4 ± 23.3 events/hr, *p* value 0.64; see Table 2. Random effects modeling demonstrated a MD of 0.36 events/hr [95% CI −2.05, 2.77] and overall effect *z* = 0.3 (*p* value = 0.77). There was significant heterogeneity with a *Q*-statistic *p* value < 0.00001, and high inconsistency with an *I* ^2^ = 83%. The SMD was 0.11 [95% CI −0.38, 0.60], overall effect *z* = 0.43 (*p* value 0.67), demonstrating a minimal to small effect using Cohen's guidelines (see Table 3). There was significant heterogeneity (*Q*-statistic *p* value < 0.0001) and high inconsistency with an *I* ^2^ = 76%, see Figure 1. A sensitivity analysis for both MD and SMD demonstrated that after the removal of the study by Djupesland et al., there was no heterogeneity and no inconsistency, with an *I* ^2^ = 0%. The respiratory disturbance index was reported by one study (Redline et al.) with 46 patients having a pretreatment RDI of 11.8 ± 9.6 events/hr and a treatment RDI of 9.8 ± 9.3 events/hr.

### 3.4. Apnea Index

A total of eleven studies reported AI outcomes. Ten studies comprising 190 patients had pretreatment and treatment data, with an AI M ± SD of 23.4 ± 25.7 and 21.0 ± 20.3 events/hr, *p* value = 0.31, see Table 2. Random effects modeling demonstrated a MD of −0.01 events/hr [95% CI −2.01, 1.99], overall effect *z* = 0.01 (*p* value = 0.99). There was no statistically significant heterogeneity (*Q*-statistic 0.34) and minimal to low inconsistency, with an *I* ^2^ = 12%. The SMD was −0.06 [95% CI −0.28, 0.15], overall effect *z* = 0.58 (*p* value 0.56), see Table 3. There was no statistically significant heterogeneity (*Q*-statistic *p* value = 0.39) and minimal inconsistency (*I* ^2^ = 7%). The funnel plot was clustered near the zero values, suggesting a risk of bias.

### 3.5. Lowest Oxygen Saturation

Ten studies with 208 patients reported LSAT outcomes and had pretreatment and treatment data for LSAT, with a M ± SD of 78.7 ± 13.4 and 79.1 ± 12.4%, *p* value = 0.75, see Table 2. Random effects modeling demonstrated a MD of 0.98% [0.09, 1.87], overall effect *z* = 2.16 (*p* value = 0.03). There was no statistically significant heterogeneity (*Q*-statistic *p* = 0.40), and there was no to minimal inconsistency (*I* ^2^ = 4%). The SMD was 0.14 [95% CI −0.10, 0.38], overall effect *z* = 1.18 (*p* value = 0.24), see Table 3. There was no significant heterogeneity (*Q*-statistic *p* value = 0.20) and low to moderate inconsistency (*I* ^2^ = 26%). The funnel plot is clustered toward the center, suggesting a risk of publication bias.

### 3.6. Scoring Criteria for Snoring

Hoijer et al. [22] measured snoring by the number of epochs with sound energy level above 55 dB and found a significant reduction in snoring. Liistro et al. [29] found no difference in snoring index with nasal dilators, but defined snoring index as the number of 30 second sleep epochs with at least one snore over the total number of sleep epochs. Metes et al. did not report methodology for snoring index calculation. Pevernagie et al. [31] calculated the ratio of the sum of all the individual snores with peak level of at least 2 dB above background noise and total sleep time and showed a significant improvement in frequency but not loudness with nasal dilators. Schonhofer et al. [26] calculated the snoring index from the number of intervals between two snores that were greater than 11 seconds but less than 60 seconds in duration, and found no objective improvement but that the majority of bed partners reported a mild reduction in snoring when their spouse used the nasal dilator. Todorova et al. [27] obtained multiple snoring variables to include snoring vibrations (maximum and mean), noise index and snoring index. They calculated snoring index as the number of snores with snoring intensity level greater than 10 dB, 15 dB, 20 dB, 25 dB, and 30 dB per hour. They found only a significant decrease in three subcategories in all the 34 snoring variables observed, which could be explained by random error rather than a true significant finding. Wenzel et al. [28] found a significant improvement in snoring index by calculating the number of snoring events per hour.

### 3.7. Outcomes for Snoring

Polysomnography-based snoring index was reported by six studies. Five studies had a combined total of 111 patients provided means and standard deviations. The combined pretreatment and treatment data, and the snoring M ± SD were 148.2 ± 268.2 and 96.3 ± 178.2 snores/hr (*p* value = 0.09), see Table 2. Random effects modeling demonstrated a mean difference of −2.5 [−10.7, 5.71], overall effect *z* = 0.60 (*p* value 0.55). There was no statistically significant heterogeneity (*Q*-statistic *p* value = 0.2), but there was a low level of inconsistency with a value of 33%. The SMD was −0.25 [95% CI −0.52, 0.01], overall effect *z* = 1.86 (*p* value = 0.06), with no statistically significant heterogeneity (*Q*-statistic *p* value 0.55) and no inconsistency (*I* ^2^ = 0%).

### 3.8. Sleepiness

Amaro et al., Redline et al. and Schonhofer et al. utilized the ESS for 85 patients and had a combined control and treatment M ± SD of 10.3 ± 5.2 and 9.5 ± 5.6, respectively (*p* value = 0.34).

### 3.9. Subanalysis: Internal vs External Nasal Dilators

Subanalysis showed that the external nasal dilators increased the AHI in 86 patients by 0.48 [−2.13, 3.09] events/hr, overall effect *z* = 0.36 (*p* value 0.72), while the internal nasal dilators reduced the AHI by 1.37 [−9.91, 7.16] events/hr, overall effect *z* = 0.32 (*p* value 0.75). Subanalysis of apnea index showed that the external nasal dilators increased the AI in 128 patients by 0.64 [−0.98, 2.27] events/hr, overall effect *z* = 0.78 (*p* value 0.44), while internal nasal dilators changed the AI in 62 patients by −4.87 [−11.94, 2.20] events/hr, overall effect *z* = 1.35 (*p* value = 0.18). Subanalysis of lowest oxygen saturation showed that the external nasal dilators improved the LSAT in 136 patients by 1.29% [0.35, 2.23], overall effect *z* = 2.69 (*p* value = 0.007), while the internal nasal dilators improved the LSAT in 72 patients by 1.47% [−2.50, 5.43], overall effect *z* = 0.72 (*p* value = 0.47). Subanalysis of snoring outcomes showed that the external nasal dilators improved snoring in 60 patients by −3.08 [−8.47, 2.32] snores/hr, overall effect *z* = 1.12 (*p* value = 0.26), while internal nasal dilators improved snoring in 51 patients by −86.54 [−241.0, 67.9] snores/hr, overall effect *z* = 1.10 (*p* value = 0.27).

### 3.10. The Effect of Disease Duration, Smoking, Alcohol, and BMI

No study reported the disease duration. Regarding BMI, Amaro et al., Liistro et al., Redline et al., and Schonhofer et al. did not stratify outcomes based on the BMI; however, they did report that the BMI values did not change during the intervention period. Regarding alcohol and tobacco, (1) Bahammam et al. instructed patients to avoid alcohol during the study, (2) Redline et al. described instructing patients to have good sleep hygiene to include no tobacco or alcohol near bedtime, (3) Schonhofer et al. instructed patients to abstain from alcohol for 1 week prior to both the baseline polysomnographic recordings and the follow-up study, and (4) Todorova et al. reported that all subjects were free of alcohol during the study.

## 4. Discussion

Although nasal dilators may subjectively improve a patient's nasal obstruction, the devices have not been demonstrated improving sleep study parameters. Therefore, the devices should not be thought of as curative for OSA but rather should be considered as adjuncts to treatment. For example, nasal dilators may reduce the pressure required for continuous positive airway pressure (CPAP) devices [32]. Since lower CPAP treatment pressures often improve CPAP use [8], it is possible that nasal dilator use may also improve CPAP use. In those patients with significant improvement in breathing with nasal dilators but who cannot tolerate the devices, site directed nasal surgery may be considered. The therapeutic implications for patients who can tolerate and do receive benefit, long-term use may be considered.

Twelve of the fourteen studies in this review showed no significant change in AHI with the use of nasal dilators [18, 19, 21, 23–31]. There was a slight reduction in AI with internal nasal dilators (−4.87 events/hr). Notably, the Djupesland et al. [19] study actually demonstrated a significant increase in AHI with nasal dilator compared to placebo. Only two studies (both using internal nasal dilators), Gosepath et al. [20] and Hoijer et al. [22], demonstrated a significant 17% reduction in AHI from 31.7 to 26.3 events/hr. In the studies that showed an improvement in obstructive events the patients were not cured of OSA. Additionally, studies did not control for body position. Like the AHI, there was only a minimal change in LSAT with nasal dilator treatment. The overall mean pretreatment and treatment LSAT values improved from 76.8 ± 13.7% to 77.1 ± 12.8%, which was not a significant improvement. These findings are consistent with the understanding that the nose generally is not considered a site of obstruction during apneic events, since the nose does not move dynamically. The more susceptible areas of collapse include the oropharynx, hypopharynx, soft palate, and epiglottis in OSA patients [21, 33].

Snoring demonstrated improvement when the overall raw data was combined, but there was only a small improvement when random effects modeling was applied. A subanalysis comparing external versus internal nasal dilators demonstrated that the external nasal dilators improved the snoring index minimally (−3.08), but the internal nasal dilators improved snoring more significantly (−86.54). It is unclear why the internal nasal dilators would decrease snoring more than external nasal dilators, but a possible explanation is variability in how the snoring index was defined and measured. The diverse calculation for snoring index in the included studies could explain the mixed improvement with nasal dilators. A uniform method for measuring snoring should be used in future studies in order to facilitate interpretation among studies.

## 5. Limitations

The inherent limitation in this systematic review is the lower quality of published studies evaluating nasal dilators. Most studies included were individual case-control or prospective case series studies, often with small sample sizes, lacking randomization and having other significant drawbacks. Therefore, in order to improve the level of evidence, additional, prospective trials with randomization are needed. As with any systematic review, it is possible despite best efforts that studies were missed in searching the literature. There may be differences in polysomnogram scoring criteria among institutions; however, these differences were not specified by the articles included in this review.

## 6. Conclusion

Although nasal dilators have demonstrated improved nasal breathing, they have not shown improvement in obstructive sleep apnea outcomes, with the exception of mild improvement in apnea index when internal nasal dilators were used.

## Figures and Tables

**Table 1:** General characteristics and quality criteria of included studies. Quality assessment of case series studies checklist from National Institute for Health and Clinical Excellence (NICE). (1) Was the case series collected in more than one center, that is, multicenter study? (2) Is the hypothesis/aim/objective of the study clearly described? (3) Are the inclusion and exclusion criteria (case definition) clearly reported? (4) Is there a clear definition of the outcomes reported? (5) Were data collected prospectively? (6) Is there an explicit statement that patients were recruited consecutively? (7) Are the main findings of the study clearly described? (8) Are outcomes stratified (e.g., by abnormal results, disease stage, and patient characteristics)?

	(1)	(2)	(3)	(4)	(5)	(6)	(7)	(8)
Amaro et al., 2012	No	Yes	Yes	Yes	Yes	No	Yes	No
Bahammam et al., 1999	No	Yes	Yes	Yes	Yes	Yes	Yes	No
Djupesland et al., 2001	No	Yes	No	Yes	Yes	No	Yes	Yes
Gosepath et al., 1999	No	Yes	No	Yes	No	No	Yes	No
Hoffstein et al., 1993	No	Yes	No	Yes	Yes	No	Yes	No
Hoijer et al., 1992	No	Yes	Yes	Yes	Yes	Yes	Yes	Yes
Kerr et al., 1992	No	Yes	No	Yes	Yes	No	Yes	No
Liistro et al., 1998	No	Yes	No	Yes	Yes	No	Yes	Yes
Metes et al., 1992	No	Yes	No	Yes	Yes	Yes	Yes	No
Pevernagie et al., 2000	No	Yes	Yes	Yes	Yes	No	Yes	No
Redline et al. 1998	No	Yes	Yes	Yes	Yes	No	Yes	Yes
Schonhofer et al., 2000	No	Yes	Yes	Yes	Yes	Yes	Yes	No
Todorova et al., 1998	No	Yes	Yes	Yes	Yes	No	Yes	Yes
Wenzel et al., 1997	No	Yes	Yes	No	No	Yes	Yes	No

**Table 2:** Demographic, polysomnographic, and sleepiness data pretreatment and during nasal dilator treatment. AI = apnea index; AHI = apnea-hypopnea index; BMI = body mass index in kg/m^2^; ESS = Epworth sleepiness scale; low O_2_ = lowest oxygen saturation; *N* = number; pts = patients; *∗* = external nasal dilators, *∗∗* = median reported, and *∗∗∗* = respiratory disturbance index.

First author, year	*N*	Age	BMI	Pre-ND AHI	ND AHI	Pre-ND AI	ND AI	Pre-ND low O_2_	ND low O_2_	Snoring index	Snoring Index
Amaro, 2012^*∗*^	12	52 ± 8	33.5 ± 4.6	38 ± 14	39 ± 15	17 ± 14	23 ± 18	79 ± 7	79 ± 6	—	—
Djupesland, 2001^*∗*^	18	51 ± 7.8	26.1 ± 3.1	8.7 ± 1.2	12.2 ± 1.7	—	—	87.5 ± 1.3	89.0 ± 1.8	—	—
Pevernagie, 2000^*∗*^	12	43 ± 2.8	25.1 ± 0.8	6 ± 1	6 ± 1	29 ± 3	23 ± 12	—	—	—	—
Schonhofer, 2000	26	54.8 ± 11.3	31.6 ± 5.7	37.4 ± 18.3	36.1 ± 20.1	20.3 ± 19.0	20.4 ± 16.2	70.7 ± 20.2	75.8 ± 11.5	39.6 ± 12.9	38.4 ± 19.7
Gosepath, 1999^*∗*^	26	52 (31–75)	—	31.7	26.3	22.8	19.8	—	—	—	—
Bahammam, 1999^*∗*^	18	46.3 ± 8.7	29 ± 7.4	8.9 ± 1.9	7.4 ± 2.1	16.7 ± 2.0	17.8 ± 2.1	—	—	—	—
Todorova, 1998^*∗*,*∗∗*^	30	36.5	25.9	3.5 (1.6, 9.1)	3.1 (1.3, 9.4)	16.5 (5.0, 46.0)	14.5 (5.0, 57.0)	—	—	89.1 (4.0, 142.5)	7.1 (0, 119.5)
Liistro, 1998^*∗*^	10	48 ± 12.1	30 ± 6.4	—	—	2.3 ± 4.1	3.5 ± 4.9	79 ± 12.8	81 ± 5.9	55.6 ± 27.9	56.9 ± 28.1
Redline, 1998^*∗*^	46	49.2 ± 10.5	32.0 ± 8.5	11.8 ± 9.6^*∗∗∗*^	9.8 ± 9.3^*∗∗∗*^	—	—	85.5 ± 9.6	86.2 ± 7.1	—	—
Wenzel, 1997^*∗*^	50	52.5 ± 5.7	30	—	—	29.1 ± 23.7	26.5 ± 23.7	74.2 ± 10.1	73.3 ± 12.1	31.4 ± 13.7	28.1 ± 14.5
Hoffstein, 1993	15	49 ± 10	36 ± 12	35.5 ± 29.8	33.9 ± 22.9	29.6 ± 52.4	24.9 ± 34.4	78 ± 18	69 ± 20	564 + 420	246 ± 342
Metes, 1992	10	—	—	46 ± 39	44 ± 40	36 ± 61	26 ± 39	71 ± 19	69 ± 19	484 ± 264	403 ± 155
Hoijer, 1992	11	47 (32–65)	—	—	—	18 ± 18.9	6.4 ± 4.1	78 ± 9.1	84 ± 3.4	—	—
Kerr, 1992	10	51 (29–68)	32 (25.9–38.9)	57.8 ± 35.8	56.9 ± 37.1	—	—	84.6 ± 1.9	84.6 ± 1.6	—	—
*Total*	*294*	*50.5 ± 8.3*	*30.0 ± 6.2*	*28.7 ± 24.0 (147 pts)*	*27.4 ± 23.3 (147 pts)*	*23.4 ± 25.7 (190 pts)*	*21.0 ± 20.3 (190 pts)*	*78.7 ± 13.4 (208 pts)*	*79.1 ± 12.4 (208 pts)*	*148.2 ± 268.2 (111 pts)*	*96.3 ± 178.2 (111 pts)*

**Table 3:** Summary for mean differences and standardized mean differences. AHI = apnea-hypopnea index, AI = apnea index, CI = confidence interval, LSAT = lowest oxygen saturation, MD = mean difference, SI = snoring index, and SMD = standardized mean difference.

Control and nasal dilator			
Treatment Data			
Random effects modeling MD	MD [95% CI]	Overall effect *z*	*p* value

AHI	0.36 [−2.05, 2.77]	0.3	**0.77**
AI	−0.01 [−2.01, 1.99]	0.01	**0.99**
LSAT	0.94% [−0.21, 2.09]	0.30	**0.77**
SI	−2.50 [−10.7, 5.71]	0.60	**0.55**

Random effects modeling SMD	SMD [95% CI]	Cohen's magnitude of effect	

AHI	0.11 [−0.38, 0.60]	Small	
AI	−0.06 [−0.28, 0.15]	Small	
LSAT	0.11 [−0.38, 0.60]	Small	
SI	−0.25 [−0.52, 0.01]	Small	
